# Intelligent identification system of gastric stromal tumors based on blood biopsy indicators

**DOI:** 10.1186/s12911-023-02324-y

**Published:** 2023-10-13

**Authors:** Shangjun Han, Meijuan Song, Jiarui Wang, Yalong Huang, Zuxi Li, Aijia Yang, Changsheng Sui, Zeping Zhang, Jiling Qiao, Jing Yang

**Affiliations:** 1 Department of the First Clinical Medical College, Gansu University of Traditional Chinese Medicine, Lanzhou, People’s Republic of China; 2 Department of General Surgery, Gansu Provincial Hospital, Lanzhou, People’s Republic of China; 3 Department of Medical Information and Engineering, Xuzhou Medical University, Xuzhou, People’s Republic of China

**Keywords:** Gastric stromal tumors, GST warning system, Blood indicators, Machine learning

## Abstract

**Background:**

The most prevalent mesenchymal-derived gastrointestinal cancers are gastric stromal tumors (GSTs), which have the highest incidence (60–70%) of all gastrointestinal stromal tumors (GISTs). However, simple and effective diagnostic and screening methods for GST remain a great challenge at home and abroad. This study aimed to build a GST early warning system based on a combination of machine learning algorithms and routine blood, biochemical and tumour marker indicators.

**Methods:**

In total, 697 complete samples were collected from four hospitals in Gansu Province, including 42 blood indicators from 318 pretreatment GST patients, 180 samples of gastric polyps and 199 healthy individuals. In this study, three algorithms, gradient boosting machine (GBM), random forest (RF), and logistic regression (LR), were chosen to build GST prediction models for comparison. The performance and stability of the models were evaluated using two different validation techniques: 5-fold cross-validation and external validation. The DeLong test assesses significant differences in AUC values by comparing different ROC curves, the variance and covariance of the AUC value.

**Results:**

The AUC values of both the GBM and RF models were higher than those of the LR model, and this difference was statistically significant (*P* < 0.05). The GBM model was considered to be the optimal model, as a larger area was enclosed by the ROC curve, and the axes indicated robust model classification performance according to the accepted model discriminant. Finally, the integration of 8 top-ranked blood indices was proven to be able to distinguish GST from gastric polyps and healthy people with sensitivity, specificity and area under the curve of 0.941, 0.807 and 0.951 for the cross-validation set, respectively.

**Conclusion:**

The GBM demonstrated powerful classification performance and was able to rapidly distinguish GST patients from gastric polyps and healthy individuals. This identification system not only provides an innovative strategy for the diagnosis of GST but also enables the exploration of hidden associations between blood parameters and GST for subsequent studies on the prevention and disease surveillance management of GST. The GST discrimination system is available online for free testing of doctors and high-risk groups at https://jzlyc.gsyy.cn/bear/mobile/index.html.

## Background

Gastric stromal tumors (GSTs) are a type of alimentary tract malignancy with low incidence arising from the gastric mesenchymal tissue [1]. The biological behavior and clinical manifestations of GSTs are benign to malignant. However, the prognosis for advanced GSTs is not optimistic and has a high risk of recurrence [2]. Recent studies have shown that the incidence of GSTs is increasing yearly [3]. The actual prevalence of GST may be higher due to its unobvious early clinical symptoms and its tendency to be overlooked as a diagnosis.

The exact etiology of GST is not well understood, but it is generally accepted that GSTs are associated with functional mutation of the c-kit (CD117) or platelet-derived growth factor receptor-alpha (PDGRFA) gene [4]. Radical surgery is the primary treatment used for early-stage GST and represents the only possible cure [2, 5]. Approximately 60% of patients with early-stage GST can be cured with surgery alone [6]. However, approximately 20–30% of GISTs have distant metastases at the time of diagnosis, commonly in the liver and abdomen [7], and their overall survival rate is only 23% [8]. Delayed diagnoses directly result in patients missing the best treatment modalities and timing, consequently reducing their survival rate. It is evident that the early diagnosis of GST is crucial for both the determination of patient treatment decisions and prognosis. The early diagnosis of GST is more difficult, and a lack of biomarkers and a final diagnosis can only be made by pathological examination and genetic testing via surgery or biopsy sampling. However, pathological examination requires the endoscopic or intraoperative acquisition of pathological tissue, which is invasive, risk of tumor spread, less desirable to patients, and more time-consuming. The diagnosis of GST in clinical practice also includes imaging techniques such as ultrasound endoscopy, computed tomography (CT) scans, and magnetic resonance imaging (MRI) scans, which can assist in determining a tumor’s size, morphology, and degree of infiltration. Enhanced abdominal CT scans are currently the most commonly used method for diagnosing primary and metastatic GST. This method helps monitor tumor progression and treatment effectiveness and is useful for tumor staging, surgery selection, and follow-up [9]. However, these scans have very limited reference value regarding different tumor types. A high rate of misdiagnosis is associated with diseases such as various types of gastric eminence lesions, gastric polyps and gastric cancer that may have the same symptoms as GST [10]. At present, there is still a lack of a simple and effective diagnosis and screening approach for GST patients, and early diagnosis of malignant-prone tumors is crucial to determine the therapeutic effect and prognosis of GST.

Several substances associated with tumorigenesis are often present in blood serum; these indicators are widely used in the auxiliary diagnosis of tumors. To date, tumor markers of the gastrointestinal tract still have no clear diagnostic value for GST. Recently, with the concept of liquid biopsies continuing to evolve, various biomarkers have received great attention in the diagnosis, prognosis, monitoring of disease progression and treatment response prediction [11]. Liquid biopsy aims to characterize tumors by applying these techniques to body fluids (mainly peripheral blood), as these biopsies are easy to perform, less invasive, and can be repeated regardless of temporal and spatial heterogeneity [12].

Liquid biopsies primarily involve the detection of peripheral blood circulating tumor cells (CTCs), circulating tumor DNA (ctDNA), and microRNAs (miRNAs) [13–15]. Some studies have also suggested that the tumor markers CEA, CA199 and CA724 may have some diagnostic utility in the detection of GIST [16–18].

Several studies have identified the relevance of selected miRNAs to GIST [19–22]. Tong et al. used a real-time quantitative RT‒PCR assay for miRNA detection, analysed 1888 miRNAs expressed in GIST samples, and screened six serum miRNAs for use as markers to discriminate benign from malignant GIST diagnosis. After validation, the sensitivity, specificity, and AUC of these markers in detecting malignant GISTs were 97%, 67%, and 0.874, respectively.

This suggests that miRNAs could be used as clinical markers to aid in the diagnosis of GIST. However, no miRNAs highly specific for GIST have been identified to date [23]. Additionally, there are few relevant studies on ctDNA, and it seems that it is not yet possible to detect the diagnostic effect of GIST by ctDNA [24, 25]. While this groundbreaking technology is gradually proving to provide more comprehensive information for cancer detection, the problem of differences in the concentration and stability of biomarkers in every individual may result in distinct sensitivity or specificity outcomes that limit practical applications of liquid biopsies. Furthermore, the low levels of body fluid biomarkers limit the widespread clinical use of liquid biopsies [26], as does the high cost of the test. Therefore, there is still a long way to go before liquid biopsies can be used on a large scale in the clinical setting.

Due to the various problems associated with biological markers and liquid biopsies, there is a need to design a novel and rational strategy that can accurately distinguish GST from healthy groups and gastric polyps. This study aimed to construct an early tumor warning system based on a combination of machine learning (ML) algorithms and routine blood, biochemical, and tumor marker tests. To the authors’ knowledge, this may be the first detailed assessment of the early diagnostic effect of GST using liquid biopsy combined with artificial intelligence. Additionally, we will further confirm the potential associations between certain blood indicators and GSTs to provide new suggestions for pathology and new candidate GST diagnostic markers. Ultimately, our goal is to develop an early GST warning or diagnostic system capable of conducting large-scale application and promotion and to have good health economics effects.

## Materials and methods

### Material sources

In total, 697 complete samples were collected from Gansu Provincial Hospital, The First Hospital of Lanzhou University, Lanzhou University Second Hospital, and the Cancer Hospital of Gansu Province in the last 10 years. Blood test data from 318 GST patients (excluding advanced GST) prior to treatment and 199 healthy individuals and 180 samples of gastric polyps were included, of which 339 (48.6%) were male and 358 (51.4%) were female. The median age of the participants was 55 years (range: 18–89). Positive samples were obtained from GST patients at the time of diagnosis using histopathological biopsy or postoperative pathological specimens, while negative samples were obtained from a population of medical examiners diagnosed with healthy or gastric polyps by gastroscopy performed by no less than two specialists in the relevant field. It is important to note that there is no clear definition of early, intermediate, or late stage for GIST, but when distant metastases occur in GIST, they are considered to be in the late stage and may not be suitable for surgical resection alone. Therefore, we screened GST patients without distant metastases as early-stage patients for inclusion in the study according to the National Comprehensive Cancer Network (NCCN) Clinical Practice Guidelines for Soft Tissue Sarcoma released in February 2020 [27]. A total of 1,352 samples from four hospitals were selected for collection, and after excluding positive samples with distant metastases, a total of 697 samples with qualified and complete data were included in this study.

There were 42 features per sample in data collected from routine blood tests, including 22 routine blood markers (HGB, RBC, WBC, NEUT, GR%, LYMPH #, LY%, PLT, MPV, MONO #, MO%, EOS, EOS%, BASO #, BASO%, RDW-SD, HCT, RDW-CV, MCV, PCT, MCH, MCHC) from the Sysmex XN-1000 device, 12 biochemical markers (TP、ALB、GLB、A/G、TBIL、DBIL、IBIL、ALT、AST、AST/ALT、ALP、GGT)from the Olympus AU5400 and Olympus AU2700 devices, and 8 tumor markers (AFP、CEA、CA125、CA199、PGI、PGII、PGI/PGII、ProGRP) from the Freedom Evolyzer 200-8 device. General information about the dataset is given in Table 1, and details about the patients, including sex, age, and the 42 blood test indicators, are given in Table 1. Following data collection, data were randomly divided into Spare data using general information, and the ratio of the cross-validation set to the test set was approximately 8:2.

**Table 1:** Detailed allocation number and specific information in the cross-validation set and external validation set

	Cross-validation set	External validation set	Total number	Age range (mean ± SD)	Male/Female
Positive samples	269	49	318	18–89(57.5 ± 12.1)	177/141
Healthy samples	173	26	199	23–79(49.0 ± 12.2)	107/92
Gastric polyps	158	22	180	18–87(56.2 ± 12.2)	55/125

All procedures were approved by Gansu Provincial Hospital. Informed consent was obtained from each participant following a verbal explanation of the study, and all processes were approved by the Ethics Committee of Gansu Provincial Hospital.

### Machine learning methods

ML is one of the most important branches of artificial intelligence research. ML aims to use computers as a tool to simulate human learning behavior by allowing computers to make effective decisions on problems, such as classification, by learning from data or previous experience. ML algorithms undergo continuous self-improvement to enable better performance. A good predictive model can be used to classify existing content into knowledge structures to effectively improve work efficiency. In this study, three algorithms, gradient boosting machine (GBM), random forest (RF), and logistic regression (LR), were chosen to build GST prediction models for comparison.

### Verification methods

The performance and stability of the models were evaluated using two different validation techniques: 5-fold cross-validation and external validation. A common and effective technique for guaranteeing the correctness of algorithms and the dependability of models is cross-validation. One of the five nonoverlapping segments of the cross-validation set served as the test set, and the other four segments served as the training set. Five times through this process, each sample was used as the test set once. As opposed to a cross-validation set, an external validation set is simply utilized to verify the model’s performance. The decision was made to divide the dataset into 5, as previous experiments that have used a large number of datasets and different ML algorithms indicated that the 5-fold division was an appropriate choice to obtain the best error estimate for the current dataset. The modelling processes were implemented based on 5-fold cross-validation, irrespective of the external validation set. The test set was used to assess the generalizability of the final model. The evaluation of classifier performance is crucial to obtain the best-performing classification model. This requires the selection of classifiers with better performance using evaluation criteria. There are several popular performance evaluation metrics, such as sensitivity, specificity, accuracy, Matthew’s correlation coefficient, and the area under the curve (AUC), that can be used to comprehensively evaluate the performance of diagnostic models. A true positive (TP), a true negative (TN), a false positive (FP), and a false negative (FN) are four parameters associated with these metrics.

1 $${\rm{Sens = TP/}}\left( {{\rm{TP + FN}}} \right)$$

2 $${\rm{Spec = TN/}}\left( {{\rm{TN + FP}}} \right)$$

3 $${\rm{ACC = }}\left( {{\rm{TP + TN}}} \right){\rm{/}}\left( {{\rm{TP + TN + FP + FN}}} \right)$$

4 $${\rm{MCC = TP \times TN - FP \times FN/}}\sqrt {{\rm{(TP + FP)(TP + FN)(TN + FP)(TN + FN)}}}$$

Receiver operating characteristic curves (ROCs) are a powerful tool for verifying the accuracy of models, using Sens as the y-axis and 1Spec as the x-axis to form a graph combining sensitivity and specificity. ROC and AUC are currently the most popular diagnostic performance evaluation criteria because they are insensitive to category imbalance and do not change significantly with changes in the proportion of positive and negative samples, even if these differences are significant. However, ROC and AUC are only suitable for binary classification problems and cannot be directly applied to multicategory problems. The use of ROC curves to represent the performance of classifiers in medical decision-making is intuitively useful, while the AUC in the range [0,1] can provide an intuitive value for assessing classifier performance. There is a general rule that the higher the AUC value is, the better the classification effect and the performance of the classifier.

### Gradient lifting algorithm

**Fig. 1:**
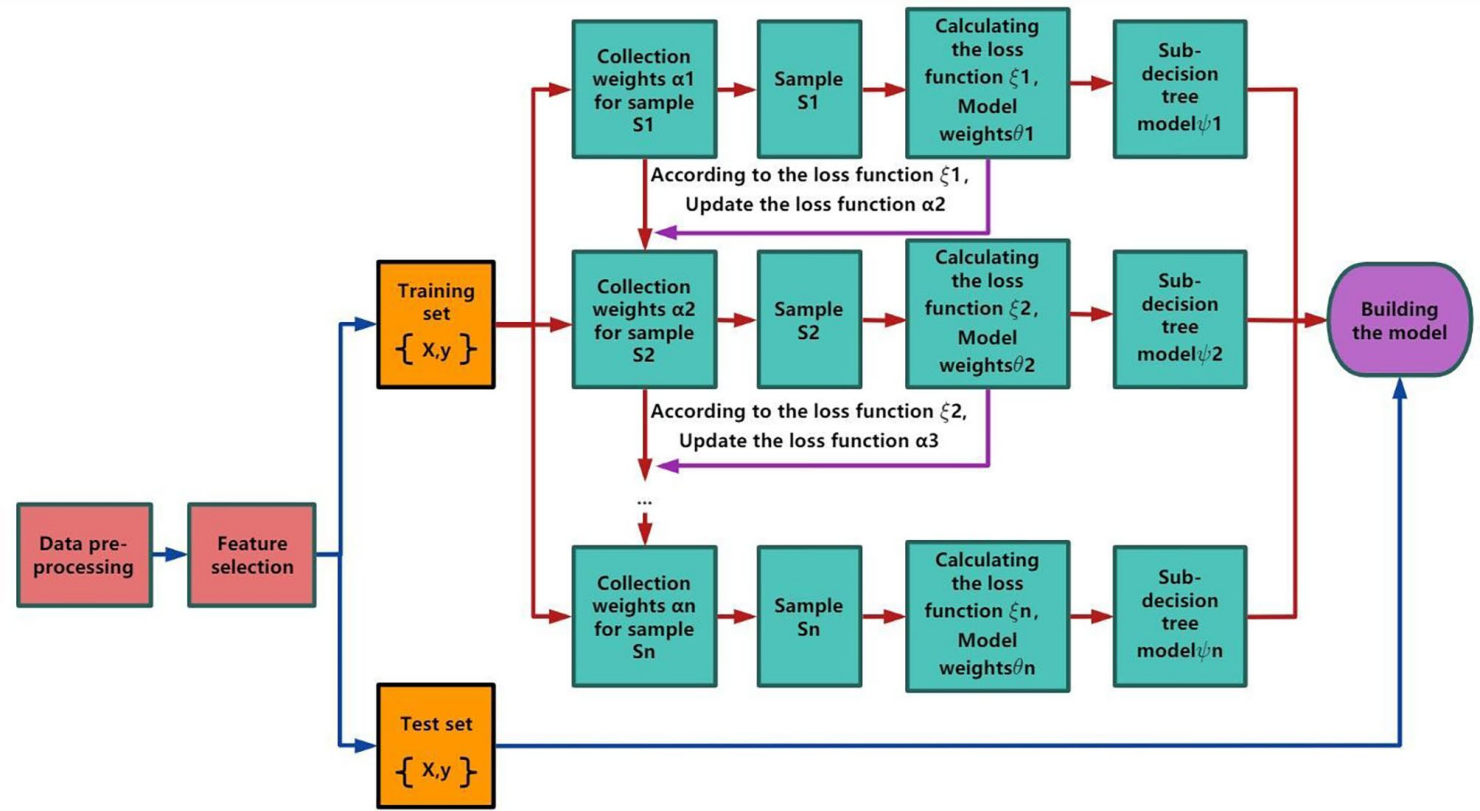
GBM modelling flow chart

The gradient lifting algorithm was proposed by Stanford professor Jerome Frideman [28]. It is mainly a decision tree-based ensemble method that trains decision trees on different labels and then combines them. The real meaning of gradient boosting machines includes gradient boosting and decision trees. First, GBM’s decision tree of choice when dealing with regression problems, binary classification, and multiclassification is the CART regression tree. A regression tree is utilized since the gradient values to be fitted to each iteration of the GBM are continuous. The most important issue is to determine the best partition point in the regression tree so that the partition point has all of the desirable values of all features. The criterion for the best dividing point in the classification tree is entropy or Gini coefficient, which is measured by purity, but the sample label in the regression tree is a continuous value, so it is no longer appropriate to use an index such as entropy instead of square error, which can judge the degree of fitting well. As shown in the flow chart of the GBM model in Fig. 1. Furthermore, to test the advantages of the gradient boosting algorithm, we used two other algorithms—random forest and logistic regression—to build prediction models simultaneously and compare the efficacy of the three models. Random forest and logistic regression have a wide range of applications in chemometrics and bioinformatics [29].

### Random forest algorithm

A random forest is a classifier that uses multiple decision trees and is built in a random manner. Multiple decision trees can be used to train and test samples, and the feature subset is randomly selected during training, which effectively reduces the occurrence of overfitting of the model and strengthens its generalization ability. The output class is determined by the mode of the output class of each decision tree. It can handle data with many features, and the features are randomly selected. In the case of unbalanced classification data, Random Forest can effectively address the errors arising from the dataset, and RF can still maintain classification accuracy even if a significant proportion of the feature data is missing.

### Logistic regression algorithm

Generalized linear regression analysis, or logistic regression, is a type of supervised learning in machine learning. Its derivation and calculation are similar to the regression process, but in fact, it is mainly used to solve the binary classification problem. The model is trained by a given set of n sets of data (the training set), and at the end of the training, the given set or sets of data (the test set) are classified. The difference from linear regression is that the input of a linear regression model is the characteristic value of the sample to be predicted, and the output is the predicted value. The logistic regression is a classification algorithm, and the output is a binary classification. The logistic regression predicts value through the sigmoid function into a probability value between 0 and 1.

## Results

### GBM diagnostic prediction model with the best performance

**Fig. 2:**
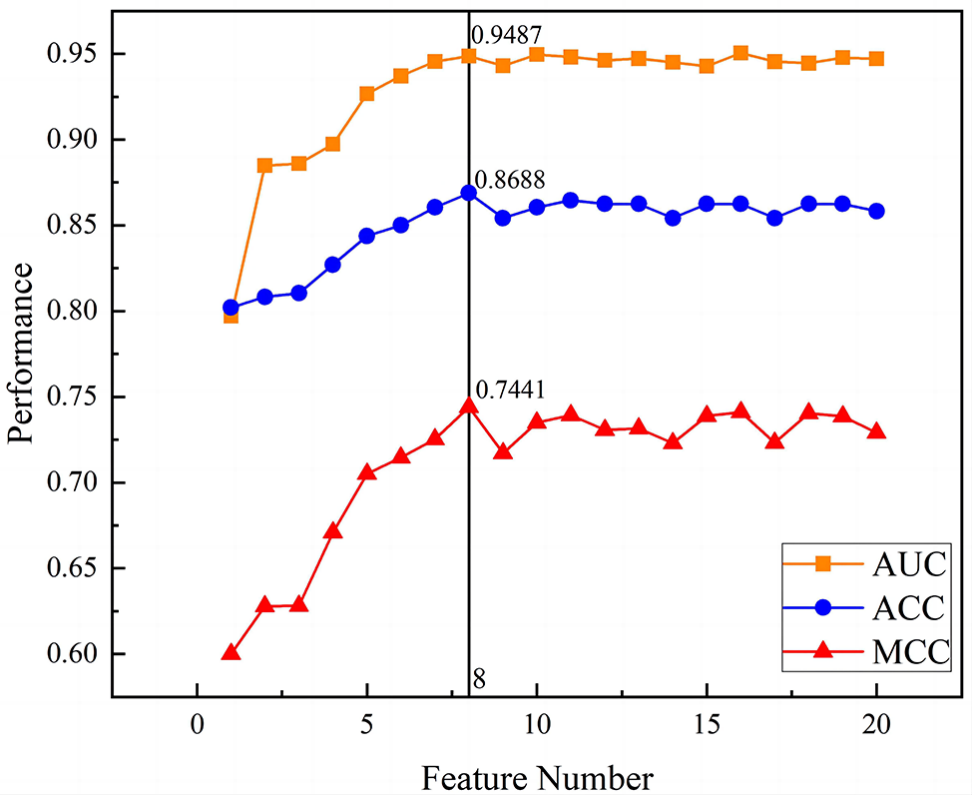
Variation in model performance with increasing number of top-ranked indices of the training set

When constructing a classification model, a reasonable selection of features helps to avoid overfitting and construct a cost-effective model. According to the principle that the minimum number of features has the same performance as all features combined, we screened a combination of eight optimal features through the Feature Importance feature screening function associated with GBM, as shown in Table 2. This feature dataset was used to construct GBM, RF, and LR prediction models. Figure 2 shows the visual representation of the feature filtering correlations, detailing the performance of the evaluated metrics for different numbers of feature combinations. Applying fewer and more appropriate feature data to construct accurate and easy-to-execute models can help simplify the blood testing process and reduce diagnostic time without compromising predictive performance.

**Table 2:** Prominent features chosen for GST discrimination

Rank	Feature(Abbreviation)	Reference range
1	Pepsinogen 1(PG-I)	70–240(ng/ml)
2	Pepsinogen 2(PG-II)	0–27(ng/ml)
3	Pepsinogen 1/Pepsinogen 2(PGI/II)	>3
4	Carbohydrate antigen125(CA125)	0–35(U/mL)
5	Albumin(ALB)	40–55(g/L)
6	Total Protein(TP)	65–85(g/L)
7	Lymphocyte(LYM%)	20–40%
8	Lymphocyte Count(LY)	1.2–3.5( 10^9/L)

“Feature importance” is a widely recognized method used to evaluate the significance of features in Gradient Boosting Machine (GBM) models, and it has garnered substantial attention in the machine learning research community. This approach facilitates the computation of importance scores for each feature within the model, which are subsequently ranked in descending order to establish a feature importance hierarchy. In the context of identifying gastric interstitial tumors for the diagnosis of stomach diseases, feature importance can be assessed through two primary perspectives: split count and split gain. Split count denotes the frequency with which a specific feature is selected as the split criterion across multiple iterations. A higher split count implies greater importance for that feature since it plays a pivotal role in the model’s decision-making process during multiple stages of the boosting process. Conversely, split gain quantifies the enhancement in the model’s objective function (e.g., reduction in squared error loss) achieved by splitting on a particular feature. A larger split gain signifies a more substantial performance improvement, indicating the heightened importance of that feature. Moreover, the insights obtained through the feature importance analysis can be invaluable for researchers and medical professionals, aiding in the identification of concealed data patterns and providing valuable guidance for both scientific investigations and practical applications. Hence, this method was employed to rank the importance of features for the detection of gastric interstitial tumors in the context of diagnosing stomach diseases, ultimately contributing to more accurate and effective diagnostic procedures.

Although we selected only the top eight features of the cross-validation set, the models achieved excellent predictive power. The GBM model was shown to be the best model, with AUC, sensitivity, and specificity values of 0.9511, 0.9411, and 0.8066, respectively.

**Fig. 3:**
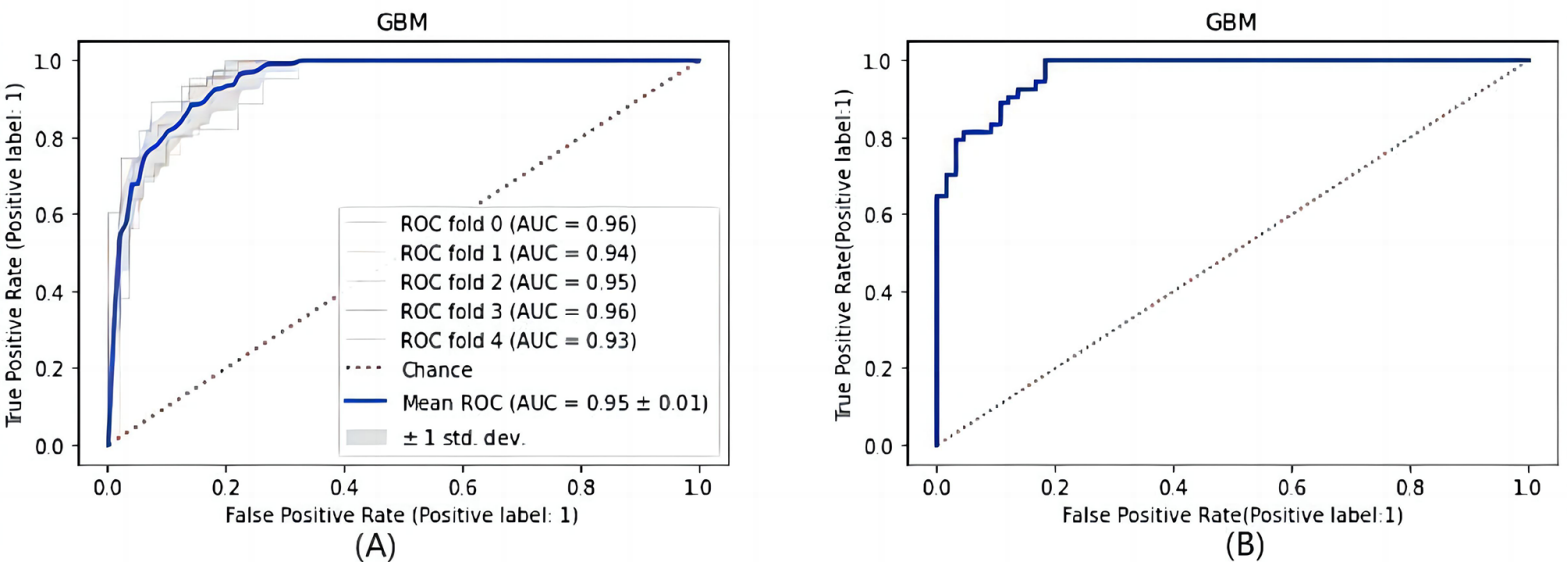
Performance of the built method in terms of the ROC curve. (**A**) ROC curve for the cross-validation set. (**B**) ROC curve for the test set

The cross-validation performance evaluations revealed that the GBM model had significant identification capability for GST patients. To more fully evaluate the GBM model, its performance was evaluated using a test set. The results remained excellent, as shown in Fig. 3. This indicates that the GBM model has strong generalizability and will demonstrate excellent adaptability to fresh samples. We also used both RF and LR algorithms to train the data and constructed prediction models based on the 5-fold cross-validation of these models; however, the results demonstrated slightly worse prediction performance for these models compared to the GBM model. The AUC values for the GBM, RF, and LR models were 0.9511, 0.9435, and 0.8121, respectively. Therefore, the GBM diagnostic model was the optimal model and had a significant ability to differentiate between patients with gastric stromal tumors and healthy individuals.

### Web server of GST discrimination to test the method

**Fig. 4:**
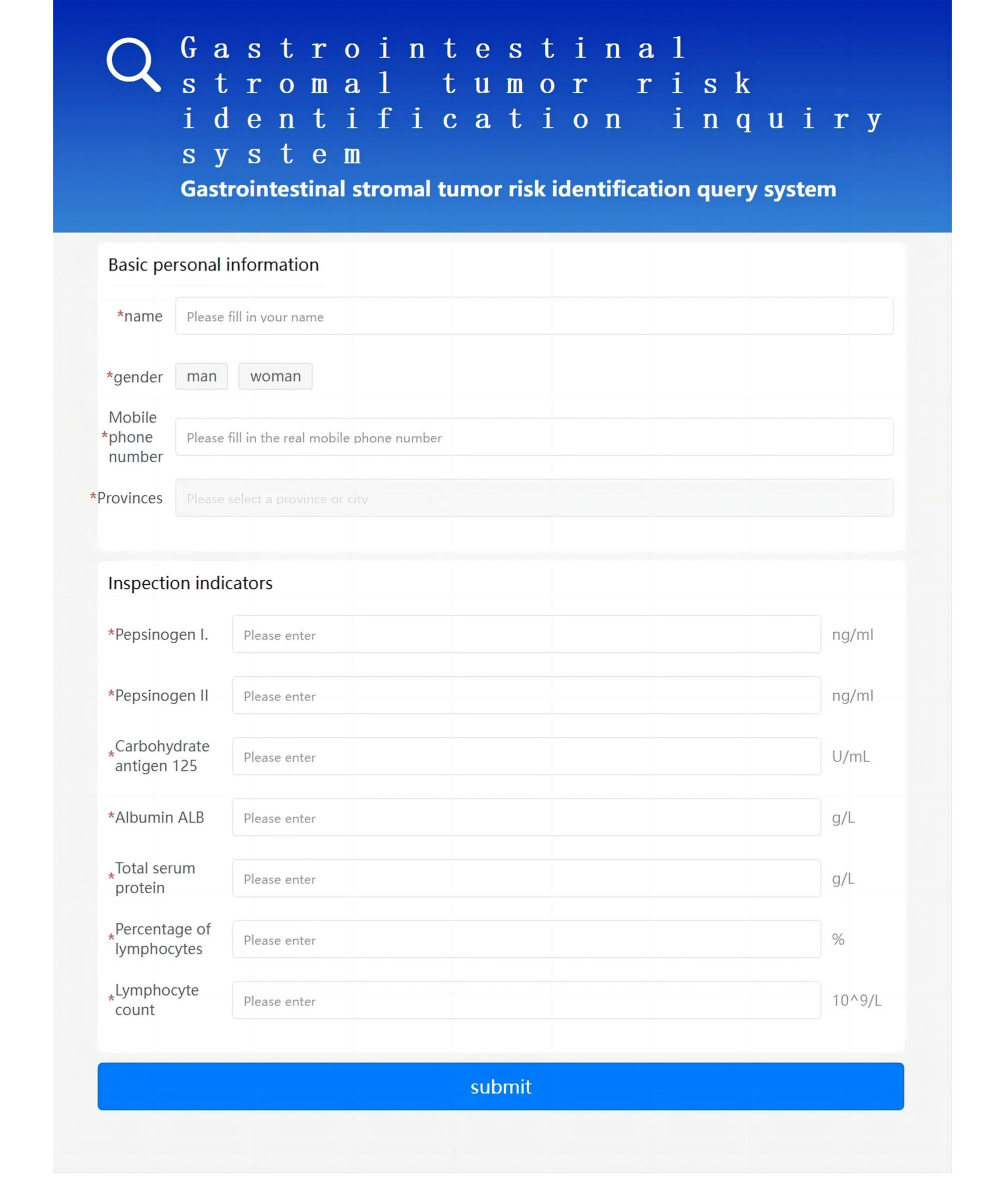
GST discrimination web server page. Users merely need to provide the indices required by the interface, and the server will display the results regarding the sample’s risk level for possible GST

**Fig. 5:**
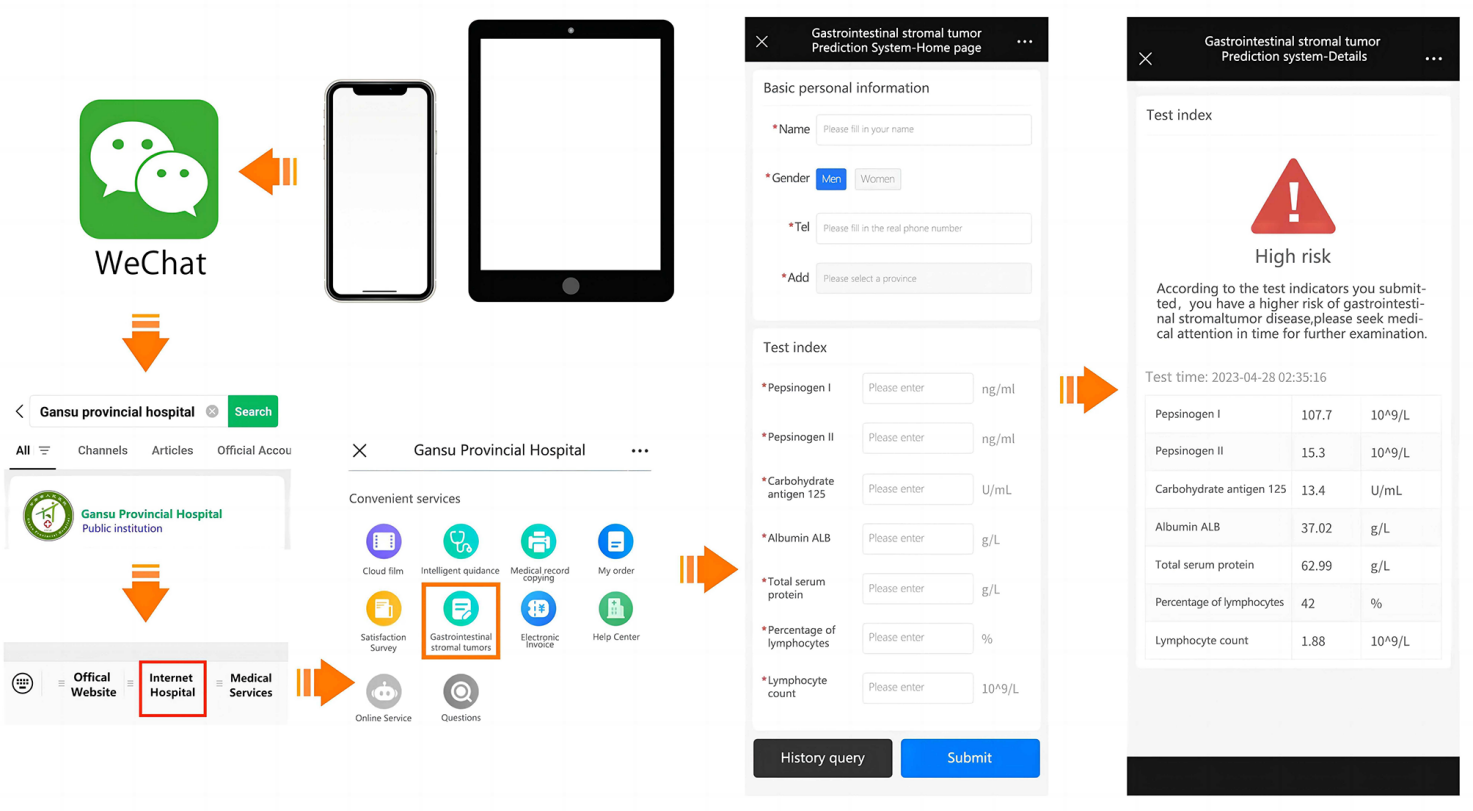
Schematic diagram of the WeChat test

At the end of this study, we designed a user-friendly online website using the GBM model, https://jzlyc.gsyy.cn/bear/mobile/index.html, accessed 10 May 2023, and set up 7 data (the PGI/II value has been hidden) input boxes to help users detect GST recognition. Moreover, we have embedded the program of this testing system in the WeChat official account of Gansu Provincial Hospital, which makes the testing system simpler and more convenient to operate. Users only need to use their mobile devices to enter the interface of the Gansu Provincial Hospital official account and find the Internet hospital interface. You can see the GIST testing icon, and click can be used. The user only needs to enter the specific 7 blood test indicators in the corresponding text box of the testing interface and then click the “Submit” button, but it is necessary to pay attention to the unit of the input value, which should be consistent with the unit behind the text box. After calculation and analysis, our model will show on the results page whether the sample belongs to a GST patient and the level of risk of having the disease. The web testing interface and WeChat official account-related interface are shown in Figs. 4 and 5. It should be highlighted that due to the possibility of systematic divergence across different devices, this identification system can only be used to assess the specific instrument utilized in this work and cannot be used to directly diagnose patients using data from numerous blood testing equipment.

## Discussion

**Fig. 6:**
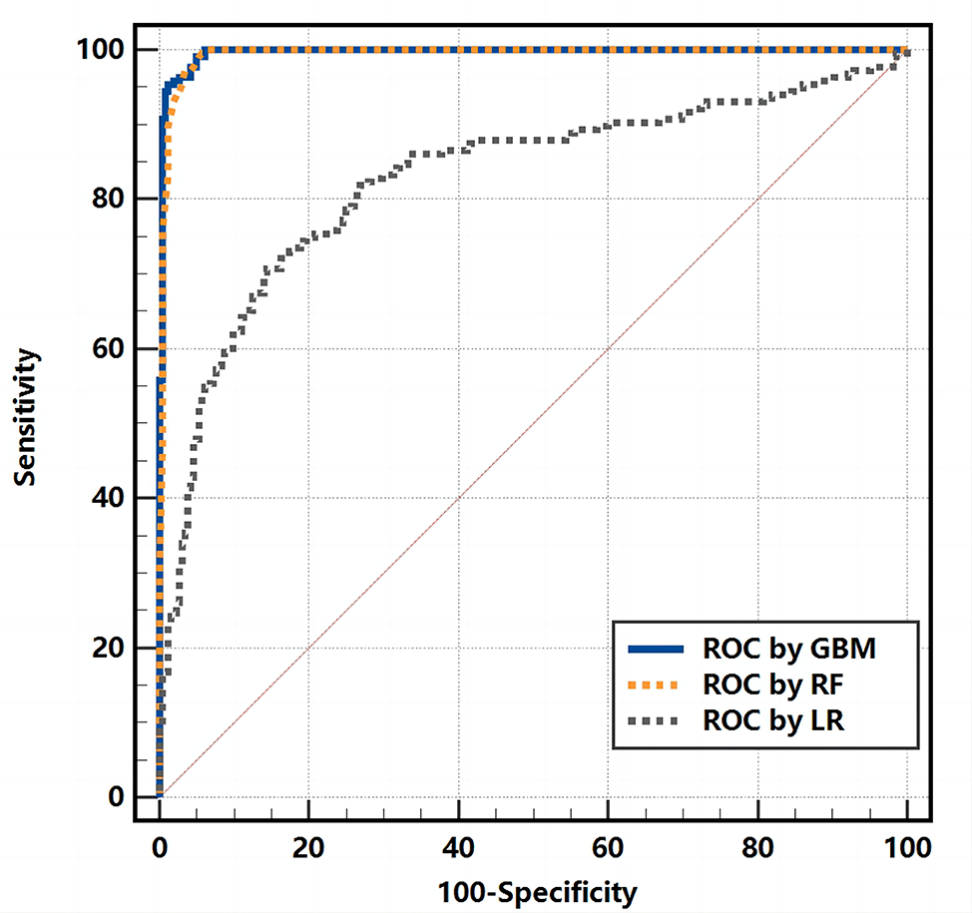
ROC curves of the three prediction models

Initial assessments concluded that both the GBM and RF models had excellent diagnostic performance and were stronger than the LR model. However, the AUC values for the three models were similar, and judging differences in model performance from this alone may not be accurate. Therefore, MedCalc 20.0 statistical software was used to perform a DeLong test on the AUC values of the three models. The DeLong test assesses significant differences in AUC values by comparing different ROC curves and the variance and covariance of the AUC value. The AUC values of two ROC curves were only considered statistically significant. Different when the P value was below 0.05. The ROC curves of the three models are shown in Fig. 6.

To verify that the model stability is reliable, the GBM model is evaluated using a test set. The results for the AUC, accuracy, sensitivity, and specificity were similarly satisfactory, at 0.9663, 0.8583, 0.9259, and 0.8030, respectively, as shown in Fig. 3B. This suggests that the discriminant system had good generalizability to newly collected blood samples and could provide clinically valuable information to assist clinicians in making diagnostic decisions. To further assess the generalizability of the method, the external validation set was completely independent of the modelling process. New blood samples were collected from The First Hospital of Lanzhou University and Gansu Provincial Hospital. In total, 49 positive and 48 negative samples were collected. The prediction results were less than satisfactory due to the small amount of external verification data, different blood test equipment and the large amount of missing key features. The assessment showed an AUC of 0.7806, an accuracy of 0.6804, a sensitivity of 0.8980, and a specificity of 0.4583. However, combining the results of the cross-validation and the performance assessment of the validation set, the GBM prediction model still appears to have potential as a simple screening and diagnostic tool for the early prediction of GST in the clinical setting.

Although pathological biopsy and histochemical analysis have long been accepted as mainstays in the diagnosis of GSTs, there are drawbacks to each. In cases of rare, insidious, and dangerous tumors such as GSTs, these drawbacks may be even more pronounced. At present, most countries do not have a health service law that provides an annual gastroscopy to screen for gastrointestinal tumors for individuals aged ≥ 40 years, although this is present in Japan [30]. As a result, various tumor markers are becoming more commonly used for the screening and initial diagnosis of various types of cancer. These markers remain very low in levels in the blood, which is a major problem for their development.

**Fig. 7:**
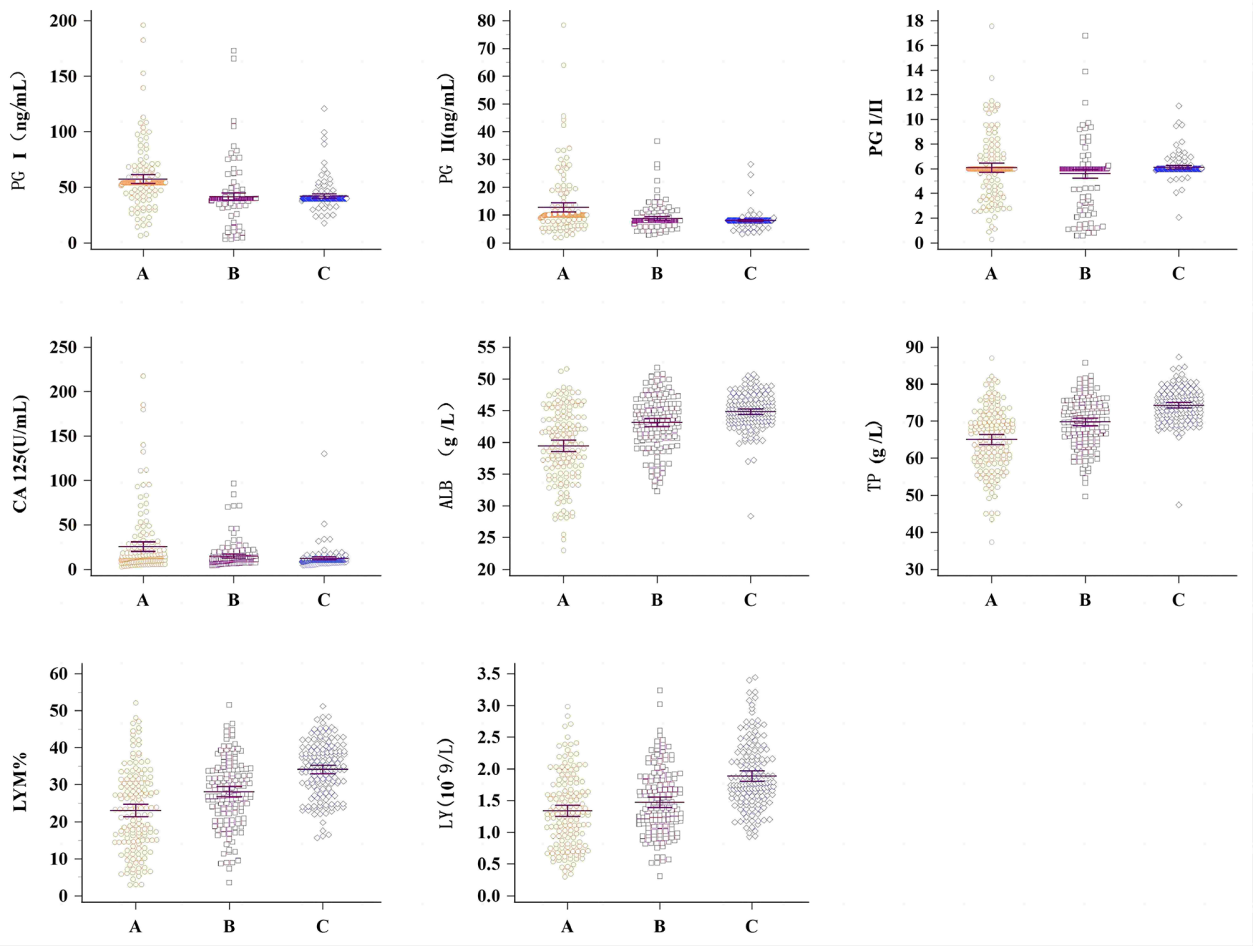
The three populations were GST group (**A**), gastric polyp group (**B**) and healthy people group (**C**)

Table 3 shows the performance of the prediction model in the current study compared with the current common GST identification methods. As a noninvasive detection technique, the results obtained in this study are no less impressive, and the prediction model is more convenient, rapid and efficient in comparison. Our research approach, using ML algorithms in combination with conventional blood test indicators, has revealed a potential link between disease and blood-related indicators, in addition to achieving excellent and robust predictive performance. We found significant variability for each of the eight chosen features in all three sample types using the Kruskal‒Wallis H-test and post hoc analysis for multifactorial independent sample comparisons (P < 0.05). As shown in Fig. 7, the expression of PGI and PGII was significantly higher in the GST group than in the gastric polyp group and the healthy group, while almost no relevant studies on the effect of pepsinogen expression on GIST could be found before this, which is a promising finding. The expression of ALB and TP, recognized as relevant proteins for monitoring nutritional status, was lower in the GST group than in the other two groups, which is consistent with other cancers. Serum CA125 is elevated in a variety of tumors and plays a key role in the diagnosis of gastrointestinal malignancies [31], but it has not received widespread attention and use in the diagnosis of GIST. Numerous studies have confirmed the correlation between the neutrophil-to-lymphocyte ratio (NLR) in cardiovascular diseases, infections, inflammatory diseases and cancer [32, 33], but no study has yet singled out LYM% or a potential association between LY and GIST, which is noteworthy.

**Table 3:** Evaluation of our solution’s performance versus commonly used GST discrimination techniques

Prediction method	Sample number	Sens (%)	Spec (%)	AUC	ACC
This work	697	0.928	0.856	0.953	0.855
CEA(16)	104	0.805	0.561	0.734	0.683
CA-199(16)	104	0.512	0.805	0.634	0.658
CEA+CA-199(16)	104	0.927	0.488	0.752	0.707
CA724(18)	123	0.815	0.548	0.79	0.572
CEA+CA-199+CA724(18)	123	0.827	0.595	0.84	0.591
CD117(17)	124	0.989	0.800	0.945	0.894
DOG1(17)	124	0.968	0.710	0.895	0.839
PDGFRA(17)	124	0.981	0.343	0.748	0.662

Therefore, the GBM model could provide valuable insights for the diagnosis of GST. More importantly, it utilizes only a small amount of clinical blood test data, which is not only simple and easy to use but also inexpensive. Of course, the current study also has some limitations in that fewer categories of negative samples were included, which may lead to the possibility that the model may also be more or less specific for other tumors. Therefore, in the follow-up, we will include gastric cancer and other GI tumor samples among the negative samples for identification with GIST and construct a comprehensive diagnostic model combining imaging histology so as to further improve the accuracy of identifying GST in a wide range of diseases.

## Conclusion

We constructed GST diagnostic prediction models using the superior classification performance of artificial intelligence ML algorithms in combination with patient blood indicators. While it reveals the potential value of conventional blood indicators, it also shows the feasibility of using the GST identification system in clinical practice. There is an opportunity to develop a new complementary diagnostic system based on this study that could provide valuable insights to clinicians in the early screening and diagnosis of GST to enable the timely and appropriate provision of treatments to improve patient outcomes. For example, before the patient needs gastroscopy and histological testing, an initial assessment can be performed. Alternatively, this predictive model can be utilized in the routine medical examination of large populations. This would help many more patients with early GST.

## Data Availability

and codes. Datasets and codes for the study are available from the corresponding author upon reasonable request with a signed agreement for scientific research purposes only.

## Abbreviations

GSTs: Gastric stromal tumors
GIST: Gastrointestinal stromal tumors
HGB: Hemoglobin
RBC: Red blood cell count
WBC: White blood cell count
NEUT#: Neutrophil count
GR%: Granulocyte ratio
LYMPH#: Lymphocyte count
LY%: Lymphocyte ratio
PLT: Platelet
MPV: Mean platelet volume
MONO#: Monocyte count
MO%: Monocyte ratio
EO#: Eosinophil count
EO%: Eosinophil ratio
BASO#: Basophil count
BASO%: Basophil ratio
RDW-SD: Standard deviation in red cell distribution
HCT: Hematocrit
RDW-CV: Coefficient variation of red blood cell volume distribution width
MCV: Mean corpuscular volume
PCT: Thrombocytocrit
MCH: Mean corpuscular hemoglobin
MCHC: Mean corpuscular hemoglobin concentration
TP: Total protein
ALB: Albumin
GLOB: Globulin
A/G: Albumin/globulin
TBIL: Total bilirubin
DBIL: Direct bilirubin
ALT: Alanine aminotransferase
AST: Aspartate aminotransferase
ALP: Alkaline phosphatase
GGT: γ-glutamyl transpeptidase
AFP: α-fetoprotein
CEA: Carcinoembryonic antigen
CA125: Carbohydrate antigen 125
CA199: Carbohydrate antigen 199
PG I: Pepsinogen I
PG II: Pepsinogen II
ProGRP: Progastrin releasing peptide
PDGFRα: Platelet-derived growth factor receptor α
CT: Computed Tomography
MRI: Magnetic resonance imaging
PET-CT: Positron Emission Tomography
CD117: Cluster of differentiation 117
DOG1: Delay of Germination 1
KIT: KIT proto-oncogene receptor tyrosine kinase
PDGFRA: Platelet-derived growth factor receptor
AUC: Area Under Curve
ACC: Accuracy
MCC: Matthews correlation coefficient
TP: True Positive
TN: True Negative
FP: False Positive
FN: False Negatives
GBM: Gradient Boosting Machine
CART: Classification And Regression Tree
RF: Random Forest
LR: Logistic regression
NGS: Next-generation sequencing
PCR: Polymerase chain reaction
GI: Tumor Digestive Tract Tumor
